# Ulceration of the Auricle of the Heart

**Published:** 1829-10-01

**Authors:** 


### Ulceration of the Left Auricle of the Heart.

Rupture of the heart, though sufficiently rare, has yet occurred so frequently as not to be a subject of much surprise on the parts of those who are best conversant with morbid anatomy. It may follow the formation of a false aneurismal sac, or it may occur, as Dr. Baillie has described, from the thinning of a part of the organ in persons advanced in life, or even the healthy heart may burst from violent emotions of the mind or exertions of the body. Dr. Whytt in 1788, and Dr. Farr more lately, in his Journal of Morbid Anatomy, have related a case in which this lesion of the organ of the circulation took place from excessive grief, and proved that "to die of a broken heart" is not a fond fiction of the fabulist or dream of the imagination, but a strictly verified pathological fact. The case that we purpose to notice, however, is not, as far as we are aware, by any means a common one, for the heart had not simply given way, but an ulcer was found in the wall of the left auricle. No notice is taken of such an occurrence in Baillie's work, nor do we remember at this moment to have read of it elsewhere. The case is recorded by M. Maréjouls in the number of the Révue Médicale for March of the present year, from which we extract the particulars.

Pierre Lemousi de Tendols, an old soldier, but then a servant, and leading a regular life, had complained for some days of pain in the left side, accompanied with cough and dyspnœa. On the second day of his illness he kept his bed, but feeling himself better on the third he rose early, and after taking breakfast, set out to walk about a league. He arrived at his journey's end completely exhausted, and the bye-standers who remarked his condition, suddenly observed him fall from his seat. They ran to his assistance, but the only sign of life he gave was a vain attempt to swallow some brandy which was offered him, when the died as instantaneously as if he had been struck by lightning. The author of the case was informed of the event, and obtained permission to open the body, which he did next day, December 31st, in the presence of M. Valantin. Previously to this he ascertained that Tendols had been ailing for fifteen or sixteen months, being oppressed by the shortest walk, and fatigued by the least exertion.

*Sectio Cadaveris.* The face was much contracted; the body extremely muscular; in the right groin was the cicatrix of an old bubo. The left pleural cavity was so filled with yellow serum that the lung was quite flattened against the vertebral column. There was no injection of the pleura, no flakes of lymph, nor any false membrane in its cavity, and the lung itself was healthy and crepitating, though fuller of blood than usual.

The pericardium, which was much distended, was opened on its anterior part when a quantity of reddish serum escaped and disclosed an immense coagulum of blood covering the entire surface of the heart. Its weight at a rough guess was estimated at two pounds. The clot having been removed, and the pericardium cleared of the blood it contained, the heart was found to be enlarged, about half as big again as the patient's fist; softer than it should be; and the parietes of its ventricles, especially the left, flabby, and extremely weak. At its upper and posterior part was a small and elongated clot, leading into the left auricle, by a rounded opening sufficiently free to allow of the introduction of the little finger. The auricle was laid open and its contents removed, when the perforation was carefully examined. It was situated at the posterior part of the auricle, a little to the left of the entrance of the right pulmonary vein; it is described to have been an "ulceration;" the lining auricular membrane was white and shining, except immediately around the ulcer, where its colour was violet red; the walls of the auricle were lacerable at this part, and evidently softened; and the appendix presented the appearance of ecchymosis. The valves were sound —the pulmonary and aortic vessels were free from morbid change, and presented no redness of their lining membrane. The stomach was filled with half digested food, and its mucous membrane was much injected. No other appearance worthy of note is described by the author of the case, but the head was not examined.

M. Maréjouls has appended no remarks to his statement, and as the case can only be of value in a pathological point of view we do not imagine that any were necessary. The history of the patient's ailments is far too meagre to allow us to pin upon it any thing like a ratio symptomatum.

